# Increased biomass accumulation in maize grown in mixed nitrogen supply is mediated by auxin synthesis

**DOI:** 10.1093/jxb/erz047

**Published:** 2019-02-13

**Authors:** Peng Wang, Zhangkui Wang, Qingchun Pan, Xichao Sun, Huan Chen, Fanjun Chen, Lixing Yuan, Guohua Mi

**Affiliations:** 1 Department of Plant Nutrition, College of Resources and Environmental Sciences, China Agricultural University, Beijing, China; 2 National Key Laboratory of Crop Genetic Improvement, Huazhong Agricultural University, Wuhan, China

**Keywords:** Ammonium, auxin, carbon and nitrogen metabolism, leaf area, maize, mixed N form, nitrate, root growth, shikimic acid pathway, source–sink relationship

## Abstract

The use of mixed nitrate and ammonium as a nitrogen source can improve plant growth. Here, we used metabolomics and transcriptomics to study the underlying mechanisms. Maize plants were grown hydroponically in the presence of three forms of nitrogen (nitrate alone, 75%/25% nitrate/ammonium, and ammonium alone). Plants grown with mixed nitrogen had a higher photosynthetic rate than those supplied only with nitrate, and had the highest leaf area and shoot and root biomass among the three nitrogen treatments. In shoot and root, the concentration of nitrogenous compounds (ammonium, glutamine, and asparagine) and carbohydrates (sucrose, glucose, and fructose) in plants with a mixed nitrogen supply was higher than that with nitrate supply, but lower than that with ammonium supply. The activity of the related enzymes (glutamate synthase, asparagine synthase, phosphoenolpyruvate carboxylase, invertase, and ADP-glucose pyrophosphorylase) changed accordingly. Specifically, the mixed nitrogen source enhanced auxin synthesis via the shikimic acid pathway, as indicated by the higher levels of phosphoenolpyruvate and tryptophan compared with the other two treatments. The expression of corresponding genes involving auxin synthesis and response was up-regulated. Supply of only ammonium resulted in high levels of glutamine and asparagine, starch, and trehalose hexaphosphate. We conclude that, in addition to increased photosynthesis, mixed nitrogen supply enhances leaf growth via increasing auxin synthesis to build a large sink for carbon and nitrogen utilization, which, in turn, facilitates further carbon assimilation and nitrogen uptake.

## Teaser

Maize plants grown in the presence of a mixed nitrate and ammonium supply exhibited rapid biomass accumulation associated with increased auxin biosynthesis and up-regulation of auxin-responsive transcripts, including those known to regulate cell expansion.

## Introduction

Plant growth is regulated by the balance of carbon (C) assimilation (source) and utilization (sink) (Krahmer *et al.*, 2018). Sufficient carbohydrate supply can promote the growth of sink organs or the formation of new sinks. Sink size or activity determines carbohydrate utilization and therefore has a feedback effect on source activity. When the carbohydrate supply exceeds the sink’s demand, the surplus carbohydrate is stored (in the form of starch, trehalose etc.) to reduce the negative feedback effect of excessive carbohydrate on photosynthesis in source leaves (Burnett *et al.*, 2016).

Nitrogen (N) assimilation needs a supply of energy and C skeletons and therefore has a fundamental effect on the C source–sink relationship (Tegeder and Masclaux-Daubresse, 2018). The coordination of C and N metabolism and utilization has a huge effect on plant growth (Prinsi *et al.*, 2018; Wang *et al.*, 2018). In most cases, nitrate (NO_3_^–^) and ammonium (NH_4_^+^) are the main forms of inorganic N in the soil. Interestingly, a mixed supply of NO_3_^–^ and NH_4_^+^ has been shown to maximize plant growth compared with a sole NO_3_^–^ or NH_4_^+^ supply (Guo *et al.*, 2007). However, the underlying physiological mechanism is not well understood. The assimilation characteristics are quite different when N is supplied as NO_3_^–^ or NH_4_^+^ (Franklin *et al.*, 2017). A deep understanding of the metabolic characteristics in the presence of a mixed N supply may provide clues. NO_3_^–^ is mainly reduced in leaves, utilizing C from local photosynthates. NO_3_^–^ reduction can compete with CO_2_ assimilation (photosynthesis) for the reducing equivalents and ATP (Marschner, 2011). The NH_4_^+^ from NO_3_^–^ reduction is assimilated via the glutamine synthase–glutamate synthase (GS-GOGAT) pathway in the chloroplasts of mesophyll cells. When NH_4_^+^ is supplied, it is mostly assimilated in the root, which requires a large amount of C transport from shoots to roots and causes an increase in the content of Gln and Asn (Pasqualini *et al.*, 2001; Hachiya *et al.*, 2012; Masakapalli *et al.*, 2013; Sato and Yanagisawa, 2014). In this process, NH_4_^+^ may promote glycolytic processes in which pyruvate kinase (PK) is activated, and a large amount of pyruvate formed from phosphoenolpyruvate (PEP) is provided to the tricarboxylic acid cycle to produce 2-oxoglutarate for NH_4_^+^ assimilation (Masakapalli *et al.*, 2013). However, super-optimal NH_4_^+^ supply can lead to the inhibition of plant growth in a process of so-called NH_4_^+^ toxicity. High NH_4_^+^ supply can cause ionic imbalance, intracellular pH disturbance, low sugar levels, and efflux of a large amount of NH_4_^+^ which leads to high root respiration and poor root growth (Britto *et al.*, 2001; Guo *et al.*, 2007, Li *et al.*, 2014). Excessive C transport from shoots to roots for NH_4_^+^ assimilation will reduce C availability for shoot growth (Britto *et al.*, 2013). Without enough sugar to satisfy both this process and root cell respiration, NH_3_ formed in the cell may become a toxic agent for respiration and cause cell death (Ganmore and Kafkafi., 1983; Esteban *et al.*, 2016). Depending on the experimental conditions—such as N concentration, pH control of the growth solution, potassium supply, and light intensity, as well as the plant genotypes being investigated—the use of sole NH_4_^+^ supply may either enhance (maize, Warncke and Barber, 1973; Li and Wang, 1993; blueberry, Claussen and Lenz, 1999; sweet pepper, Xu *et al.*, 2001; rice, Qian *et al.*, 2004) or inhibit (tobacco, Walch-Liu *et al.*, 2000; maize, Prinsi *et al.*, 2018) plant growth.

Apart from functioning as a C skeleton for N assimilation, sugars can act as signaling molecules regulating C and N metabolism in the plant. Recently, it has been found that trehalose hexaphosphate (T6P) is the precursor of trehalose responses to sucrose and glucose levels in the plant, and is induced by free NO_3_^–^ (Gazzarrini and Tsai, 2014; Yadav *et al.*, 2014). T6P can regulate plant growth and development by promoting nitrate reductase or phosphoenolpyruvate carboxylase (PEPCase) activity, inducing malic acid and oxaloacetate formation (Figueroa *et al.*, 2016), activating AGPase to promote the synthesis of starch, and inhibiting SnRK1 activity in the tissues (Gazzarrini and Tsai, 2014; Yadav *et al.*, 2014; Figueroa *et al.*, 2016). Although there are large differences in C and N metabolism under different forms of N supply, it remains unclear whether the T6P pathway is modified by these different N forms.

N metabolism has a great effect on hormone synthesis, and thus exerts profound influence on organ morphogenesis and growth. Tobacco grown under conditions of NH_4_^+^ as the sole form of N supply has lower cytokinin (CTK) levels and poorer leaf growth compared with plants supplied solely with NO_3_^–^ (Walch-Liu *et al.*, 2000). PEP is the precursor for aromatic amino acids. PEP and D-erythrose 4-phosphate form tryptophan (Trp) via the shikimic acid pathway, leading to further synthesis of auxin (IAA) via several pathways (Maeda and Dudareva, 2012). IAA biosynthesis, transport, and accumulation has been shown to be altered in response to different N regimes in maize (Tian *et al.*, 2008), soybean (Caba *et al.*, 2000), pineapple (Tamaki and Mercier, 2007), and *Arabidopsis thaliana* (Ma *et al.*, 2014). However, it remains unclear how the mixed N supply regulates hormone levels via changes in C and N metabolism.

Maize growth is strongly enhanced by the provision of N as a mixed supply of NO_3_^−^ and NH_4_^+^ (George *et al.*, 2016; Wang *et al.*, 2018). To understand the underlying physiological mechanism, we used metabolomics and RNA-sequencing (RNA-Seq) tools to reveal the changes in key metabolites and plant hormones under a mixed NO_3_^−^ and NH_4_^+^ supply in comparison to sole NO_3_^−^ or sole NH_4_^+^ supply. We hypothesized that the mixed N supply could modify metabolic pathways, improve the source–sink relationship, and therefore promote plant growth.

## Materials and methods

### Experimental procedures

Hydroponic experiments were conducted in a growth chamber with light intensity of 400 μmol m^−2^ s^−1^, day/night temperature of 28/22 °C, and 60% relative humidity. Seeds of the maize hybrid ZD958 were sterilized by treatment with 10% (v/v) H_2_O_2_ for 30 min, rinsed with deionized water, and soaked in saturated CaSO_4_ for 6 h, then transferred on to filter paper to germinate in dark conditions. When the primary root was 1.5 cm long, the seeds were transferred to culture in rolled papers. When the seedlings had one expanded leaf, the endosperm was removed and seedlings were transferred to a container (55 cm × 45 cm × 35 cm). Plants were supplied with modified Hoagland nutrient solution containing 0.5 mmol l^−1^ K_2_SO_4_, 0.6 mmol l^−1^ MgSO_4_·7H_2_O, 0.3 mmol l^−1^ KH_2_PO_4_, 0.5 mmol l^−1^ CaCl_2_·2H_2_O, 1 µmol l^−1^ H_3_BO_3_, 0.5 µmol l^−1^ MnSO_4_·H_2_O, 0.5 µmol l^−1^ ZnSO_4_·7H_2_O, 0.2 µmol l^−1^ CuSO_4_·5H_2_O, 0.07 µmol l^−1^ Na_2_MoO_4_·2H_2_O, and 0.1 mmol l^−1^ Na-Fe-EDTA. In our preliminary experiment, plant growth was highest at a NH_4_^+^ to NO_3_^−^ ratio of 75%/25% and the optimum N concentration was 1 mmol l^−1^. Therefore, N was supplied at 1 mmol l^−1^, with three different NO_3_^−^/NH_4_^+^ ratios (NO_3_^–^ alone, 75%/25% NO_3_^–^/NH_4_^+^, and NH_4_^+^ alone ) using KNO_3_ and/or (NH_4_)_2_SO_4_. MgSO_4_ and K_2_SO_4_ were added to balance differences in potassium in the solutions (Gu *et al.*, 2013). The solution pH was adjusted to 5.8 every 6–12 h. The containers were randomly placed and their positions were changed frequently. The nutrient solution was aerated continuously and renewed every 3 days.

### Biomass, leaf area, photosynthetic rate, and C and N concentration

Five seedlings from each treatment were sampled 12 days after transplanting. Leaf length and width were measured with a ruler and leaf area was calculated as length × width × *k* (where *k* is 0.75 if a leaf is fully expanded and is 0.5 if a leaf is not fully expanded; Gallais *et al.*, 2006). The most recent fully expanded leaf was used to measure photosynthetic rate by using a portable photosynthesis system (Li6400; LI-COR, Lincoln, NE, USA) coupled to a standard red/blue LED broadleaf cuvette (6400-02B; LI-COR) and a CO_2_ mixer (6400–01; LI-COR) at a light intensity of 400 μmol m^–2^s^–1^. Measurements were obtained at a leaf temperature of 28±0.5 °C and a CO_2_ concentration inside the chamber of 400±1 µmol l^–1^. The shoot and root of each seedling were separated and dried in an oven at 70 °C until the weight was unchanged, and the weight of the fully dried shoot and root was used as a measure of plant biomass. Each biological replicate consisted of one seedling. Milled dry shoot and root samples (80 mg) were used to measure C concentration using an N/C analyzer (vario MACRO cube; Elementar, Germany). There were five biological replicates for each treatment.

### Non-target metabolites and plant hormones

Fresh plant samples (100 mg) were transferred into 5 ml centrifuge tubes, five steel balls were added and then placed into liquid N for 5 min. Tubes were placed in a high flux organization grinding apparatus (70 Hz 1 min); 1000 μl of methanol (pre-cooled at –20 °C) was added and the mixture was vortexed for 30 s. The tubes were placed into an ultrasound machine at room temperature for 30 min. Next, 750 μl chloroform (pre-cooled at –20 °C) and 800 μl deionized water (4 °C) were added, tubes were vortexed for 60 s and then centrifuged for 10 min at 16 000 × *g* at 4 °C. A 1 ml aliquot of the supernatant was transferred into a new centrifuge tube. Samples were dried by vacuum concentration. Samples were then dissolved in 250 μl methanol aqueous solution (1:1) at 4 °C and filtered through a 0.22 μm membrane filtration to produce samples ready for liquid chromatography–mass spectrometry (LC-MS) detection. For quality control samples, 20 µl was taken from each prepared sample extract and mixed; the remainder of the samples was used for LC-MS.

Chromatographic separation was accomplished in a Shimadzu LC-30A system equipped with an ACQUITY UPLC^®^ HSS T3 (150 × 2.1 mm, 1.8 µm, Waters) column maintained at 40 °C. The temperature of the autosampler was 4 °C. Gradient elution of analyses was carried out with 0.1% formic acid in water (A) and acetonitrile (B) at a flow rate of 0.3 ml min^−1^. Injection of 5 μl of each sample was done after equilibration. An increasing linear gradient of solvent B (v/v) was used as follows: 0–0.5 min, 2% B; 0.5–9 min, 2%–50% B; 9–12min, 50%–98% B; 12–13 min, 98% B; 13–14 min, 98%–2% B; 14–15 min, 2% B.

The electrospray ionization–mass spectrometry experiments were executed on an AB 5600+ mass spectrometer with a spray voltage of 5.50 kV and –4.50 kV in positive and negative modes, respectively. Gas1 and gas2 were both set at 50 psi. Curtain gas was 35 psi. The source temperature was 500 °C. The mass analyzer scanned over a mass range of m/z 100–1500 for full scan at the collision energy of 45 eV. Dynamic exclusion was implemented. There were seven biological replicates for each treatment.

After the end of the assay, the metabolites were confirmed on the basis of their exact molecular weights and the possible empirical formulae of the metabolites were speculated (molecular weight error <30 ppm). The exact molecular weights were then used to identify potential biomarkers by querying the Human Metabolome Database (http://www.hmdb.ca), Metlin (http://metlin.scripps.edu), massbank (http://www.massbank.jp/), and Lipid Maps (http://www.lipidmaps.org). When analyzing, we found an abnormal sample of shoots grown under the mixed N supply, and deleted their data from the data set.

For assay of the plant hormones IAA, CTK, brassinosteroid (BR), gibberellin 3 (GA3), jasmonic acid (JA), and salicylic acid (SA), fresh shoot and root samples (250 mg) placed in a centrifuge tube and 500 μl *N*-propanol-ddH_2_O-HCl (2:1:0.002 v/v/v) was added, followed by mixing and extraction for 30 min at 4 °C; then, 1 ml dichloromethane was added and the mixture was extracted for 30 min at 4 °C, followed by centrifugation at 3000 × *g* for 20 min. A 1 ml sample of the lower fluid phase was collected, concentrated by centrifugation, and then dissolved sample in 20 μl 80% methanol. After centrifugation, the sample was passed through a 0.22 μm filter. The chromatography and mass spectrometry conditions were as described by Kojima *et al.* (2009). There were seven biological replicates for each treatment. Metabolomics and hormone analysis were conducted using the Suzhou BioNovoGene Metabolomics Platform.

### mRNA library construction and sequencing

Total RNA was extracted as described by Gu *et al.* (2013). RNA fragments were reverse-transcribed to create the final cDNA library in accordance with the protocol for the mRNA-Seq sample preparation kit (Illumina, San Diego, CA, USA); the average insert size for the paired-end libraries was 300 bp (±50 bp). Paired-end sequencing was performed on an Illumina HiSeq 4000 at LC Sciences, Houston, TX USA, following the vendor’s recommended protocol.

### Bioinformatics analysis of RNA-Seq data

Raw reads were pre-processed to remove low-quality regions and adapter sequences. Transcriptome sequencing data statistics and quality evaluation are shown in Table S4 available at Dryad Digital Repository (https://doi.org/10.5061/dryad.cd57c84; Wang *et al.*, 2019). Clean reads from each sample were aligned to the maize reference genome (B73 RefGen_v3; http://www.maizegdb.org/assembly/) using TopHat2 (Kim *et al.*, 2013). Aligned reads from TopHat2 mapping were subjected to String Tie for DeNovo transcript assembly (Pertea *et al.*, 2015). The R package ‘edgeR’ was used to identify differentially expressed genes. The expression of each gene was normalized to fragments per kilobase of transcript per million reads (FPKM) to compare among different samples. The differentially expressed mRNAs and genes were selected with log2 (fold change) >1 or log2 (fold change) <–1 and with statistical significance *P*<0.05.

Gene ontology (GO) term enrichment of differentially expressed genes was conducted using the web-based agriGO software (http://bioinfo.cau.edu.cn/agriGO/analysis.php). Singular enrichment analysis was used to compute enriched categories by comparing a list of differentially expressed genes with background genes. GO terms of gene sets of interest were compared with the genome-wide background with an adjusted *P* value (false discovery rate) cutoff of 0.01. MapMan was used to show the functional categorization of differentially expressed genes in different cellular and metabolic processes (Thimm *et al.*, 2004).

### RT–PCR analysis

A 7500 Real-Time PCR System (Applied Biosystems) was used to carry out a two-step PCR procedure. The primers used in the quantitative PCR analyses are listed in Table S1 at Dryad. Among them, the DAHP synthase (GRMZM5G828182), shikimate kinase (GRMZM2G004590), and indole-3-glycerol phosphate synthase (GRMZM2G106950) genes are involved in Trp synthesis in the shikimate and Trp synthesis pathways; E3 ubiquitin-protein ligase and RING protein genes (GRMZM2G098637, GRMZM2G040803, GRMZM2G392320, GRMZM2G170413, GRMZM2G364612, GRMZM2G068239, GRMZM2G095873, GRMZM2G178038), auxin response factor *ARF* (GRMZM2G405474), and *SAUR* family member (GRMZM2G460861) genes are involved in auxin response-related pathways. The maize *ZmUbiquitin* gene was used as an internal control for normalizing gene expression in maize.

### Amino acid determination

Plant samples (50 mg) were used for measuring the concentrations of 18 amino acids by liquid chromatography. Supercritical fluid extraction of free amino acids from shoots and roots for amino acid determination was performed as described by Dai *et al.* (2014). Seven biological replicates were used per treatment.

### Sucrose, glucose, fructose, and starch determination

Plant samples (50 mg) were used for measuring the concentrations of sucrose, glucose, fructose, and starch according to the method in Arnáiz *et al.*, (2012). Six biological replicates were used per treatment.

### Measurement of PEP, OAA, and enzyme activities

Samples of fresh shoot or root (100 mg) were placed in a centrifuge tube, 900 μl 0.01 M (pH 7.3) PBS buffer was added, and the mixture was centrifuged at 1500 × *g* for 20 min. The supernatant was used to determine PEP, oxaloacetate (OAA), and the activities of PEPCase, asparagine synthase (ASNS), trehalose-phosphate synthase (TPS), trehalose-phosphate phosphatase (TPP), and ADP-glucose pyrophosphorylase (AGPase), using ELISA (Crowther, 1995). The antibodies for ELISA were provided by Shanghai Run Yu Biotechnology Co. Ltd. GS activity was determined by reference to Xue (1985). Invertase activity was assayed by the dinitrosalicylic acid method (Mansouri *et al.*, 2013) using a kit provided by Beijing Solarbio Science & Technology Co., Ltd. Six biological replicates were used for each treatment.

### Free nitrate and ammonium

For measurement of free NO_3_^–^, 100 mg samples of ground fresh shoot or root were placed in a centrifuge tube with 1 ml double distilled H_2_O, and placed in a water bath at 95 °C for 30 min. The mixture was centrifuged at 21 000 × *g* for 15 min at 4 °C, and the supernatant was collected. NO_3_^–^ in the samples was determined using a Walters H-Class UPLC and Agilent strong anion exchange column (Agilent ZORBAX SAX 5 μm 4.6 × 240 mm) and detected with a UV detector at a wavelength of 200 nm. The mobile phase was 50 mM KH_2_PO_4_-H_2_PO_4_ (pH 3.0).

For measurement of free NH_4_^+^, 100 mg samples of ground fresh shoot or root were placed in a centrifuge tube, 1 ml pre-cooled (4 °C) 10 mM formic acid was added, and the mixture was centrifuged at 21 000 × *g* for 15 min. A 24 μl aliquot of supernatant was taken and mixed with 400 μl OPA buffer [pH 6.8; 50 ml: 100 mM KH_2_PO_4_/K_2_HPO_4_ buffer with 0.201 g *o*-phthalaldehyde (OPA) and 35.2 μl β-mercaptoethanol]. A column-free Agilent 1260 HPLC with an FLD fluorescence detector was used to measure free NH_4_^+^, with an excitation wavelength of 410 nm and a collection wavelength of 470 nm.

### Statistical analysis

Data were subjected to ANOVA, performed in SPSS Statistics 19.0 (SPSS Inc., Chicage, IL, USA). Differences were compared using the least significant difference test at the 0.05 level of probability. Heat maps were produced using the R package ‘pheatmap’ and the function ‘pheatmap’.

## Results

### Plant growth

**Fig. 1.**
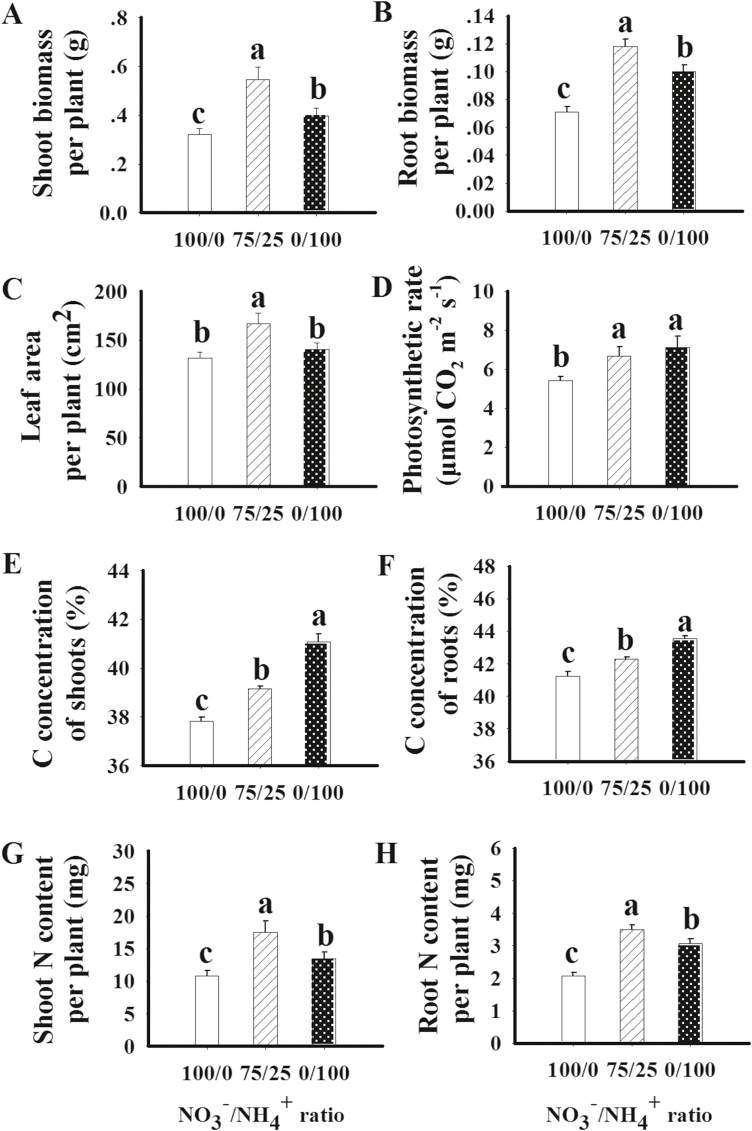
Shoot (A) and root (B) biomass, leaf area (C), photosynthetic rate (D), shoot (E) and root (F) C concentration, and shoot (G) and root (H) N content of maize plants supplied with different forms of N for 12 d. 100/0, sole NO_3_^–^ supply; 75/25, 75%/25% NO_3_^–^/NH_4_^+^; 0/100, sole NH_4_^+^ supply. Values are mean ±SE (*n*=5). Significant differences between treatments (*P*<0.05) are indicated with different letters.

In comparison to sole NO_3_^–^ supply, the mixed N supply considerably increased shoot biomass (1.70-fold), root biomass (1.66-fold), leaf area (1.27-fold), and photosynthetic rate (1.24-fold). Sole NH_4_^+^ supply slightly increased shoot biomass (1.23-fold), root biomass (1.41-fold), and photosynthetic rate (1.31-fold), but had little effect on leaf area (Fig. 1). Chlorophyll concentration was greater in the presence of mixed N and (especially) sole NH_4_^+^ supply relative to sole NO_3_^–^ supply (Fig. S1A at Dryad). The C concentration in the shoot and root was increased slightly by mixed N, and greatly by sole NH_4_^+^ (Fig. 1E, F). The mixed N supply increased the shoot and root N content of plants 1.63- and 1.70-fold, respectively, while sole NH_4_^+^ increased shoot and root N content 1.25- and 1.49-fold (Fig. 1G, H). The shoot N concentration was the same with either sole NO_3_^–^ or sole NH_4_^+^ treatment, and both were higher than in the mixed N treatment. Root N concentration was similar across the three treatments (Fig. S1B, C at Dryad).

### Metabolomics and transcriptome analysis

The effect of different N forms on metabolite profiling was investigated by LC-MS. The total ion current LC-MS chromatogram is shown in Fig. S2 at Dryad. Taking sole NO_3_^–^ supply as the control treatment, a total of 52 differential metabolites were identified under mixed N supply and NH_4_^+^ supply, with 23 in the shoot and 39 in the root (Tables S2 and S3 at Dryad) (Wang *et al.*, 2019). Principal component analysis and partial least squares discriminant analysis on the differential metabolites under the different N treatments indicated that they could be clearly distinguished into different groups (Figs S3 and S4 at Dryad).

Compared with gene expression in the presence of sole NO_3_^–^, 802 differentially expressed genes were found under mixed N supply, and 510 under sole NH_4_^+^ supply, in shoot. Among these, 152 genes were up- or down-regulated in common in both treatments. Furthermore, in root, 964 differentially expressed genes were found under mixed N supply and 971 under sole NH_4_^+^ supply; among these, 340 genes that were up- or down-regulated in common in both treatments (Fig. S5 at Dryad). GO and gene annotation analysis indicated that these genes are mainly involved in plant photosynthesis (Table S5 at Dryad), ion or nutrient absorption and transportation (Tables S6 and S7 at Dryad), amino acid and organic acid metabolism, trehalose metabolism, and auxin synthesis and the auxin response pathway (Figs S6 and S7 at Dryad).

### Key differential metabolites

**Fig. 2.**
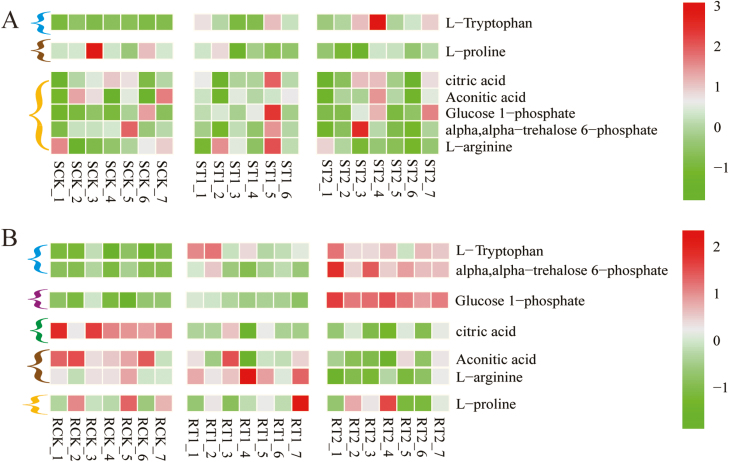
Metabolomics heat map based on LC-MS secondary mass spectrometry. The color gradient illustrates the *Z*-scores of seven metabolites in the shoot (A) and root (B), calculated as the mean-centered normalized intensity values divided by the SD for each metabolite. SCK, ST1, and ST2 indicate the metabolites in the shoots of plants exposed to sole NO_3_^–^, mixed N, and sole NH_4_^+^ supply, respectively. RCK, RT1, and RT2 indicate the metabolites in the root of plants exposed to sole NO_3_^–^, mixed N, and sole NH_4_^+^ supply, respectively. Seven repeats were performed for each N treatment. Significant difference for each metabolite was defined as *P*<0.05 and fold change ≥1.5 or ≤0.66. Compared with the effects of sole NO_3_^–^ supply, the concentration of metabolites is either increased by both mixed N and sole NH_4_^+^ supply (blue bracket), increased by only sole NH_4_^+^ supply (purple bracket), reduced by both mixed N and sole NH_4_^+^ supply (green bracket), reduced by only sole NH_4_^+^ supply (brown bracket), or does not differ among the treatments (orange bracket).

Pathway analysis using KEGG (https://www.kegg.jp/) and MetPA (www.metaboanalyst.ca) revealed that, among the 52 differential metabolites identified in the shoot and root, seven are involved in important C and N metabolic processes, including amino acid metabolism, sugar metabolism, and organic acids and related signal transduction processes. L-tryptophan is the precursor of auxin synthesis via the shikimic acid pathway. The concentration of L-tryptophan was increased by both mixed N supply and sole NH_4_^+^ supply, by 2.29- and 3.31-fold in the shoot (Fig. 2A) and 1.64- and 1.85-fold in the root (Fig. 2B). Glucose monophosphate (G1P) and T6P participate in starch and trehalose metabolism. Compared with plants exposed to NO_3_^–^ supply, G1P and G6P in the root were increased by 2.60- and 4.51-folds in plants treated with NH_4_^+^ supply. Mixed N supply increased T6P to a lesser extent (1.87-fold). Citric acid and aconitic acid are involved in respiratory metabolism in mitochondria. The different forms of N did not affect the concentration of either acid in the shoot. In the root, the concentration of citric acid was reduced by both mixed N (0.57-fold) and NH_4_^+^ (0.45-fold) supply. The concentration of aconitic acid was reduced by NH_4_^+^ supply (0.48-fold). NH_4_^+^ supply was also associated with reduced proline concentration in the shoot and L-arginine in the root (Fig. 2).

### Auxin and other plant hormones

**Fig. 3.**
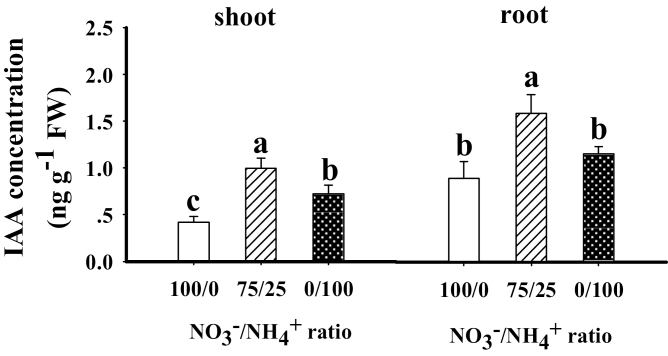
IAA concentration in shoots and roots of maize plants grown in the presence of different forms of N. 100/0, sole NO_3_^–^ supply; 75/25, 75%/25% NO_3_^–^/NH_4_^+^; 0/100, sole NH_4_^+^ supply. Values are mean ±SE (*n*=7). Significant differences between treatments (*P*<0.05) are indicated with different letters.

Compared to sole NO_3_^–^ supply, the mixed N supply increased the concentration of IAA in the shoot (2.39-fold) and root (1.77-fold). The sole NH_4_^+^ supply increased the IAA concentration in the shoot to a lesser extent (1.72-fold), but had no significant effect on the concentration in the root (Fig. 3). Considering the other plant hormones, in the shoot, the mixed N supply was associated with higher levels of BR and JA, and lower levels of CTK, GA3, and SA, compared with NO_3_^–^ supply (Fig. S8 at Dryad). The sole NH_4_^+^ treatment increased shoot BR and JA, reduced GA3, and had no effect on CTK, BR, and SA. In the root, compared with sole NO_3_^–^ supply, the mixed N treatment increased CTK, BR, JA, and SA, and had no effect on GA3; sole NH_4_^+^ supply increased CTK and SA, reduced GA3, and had no effect on BR and JA.

**Fig. 4.**
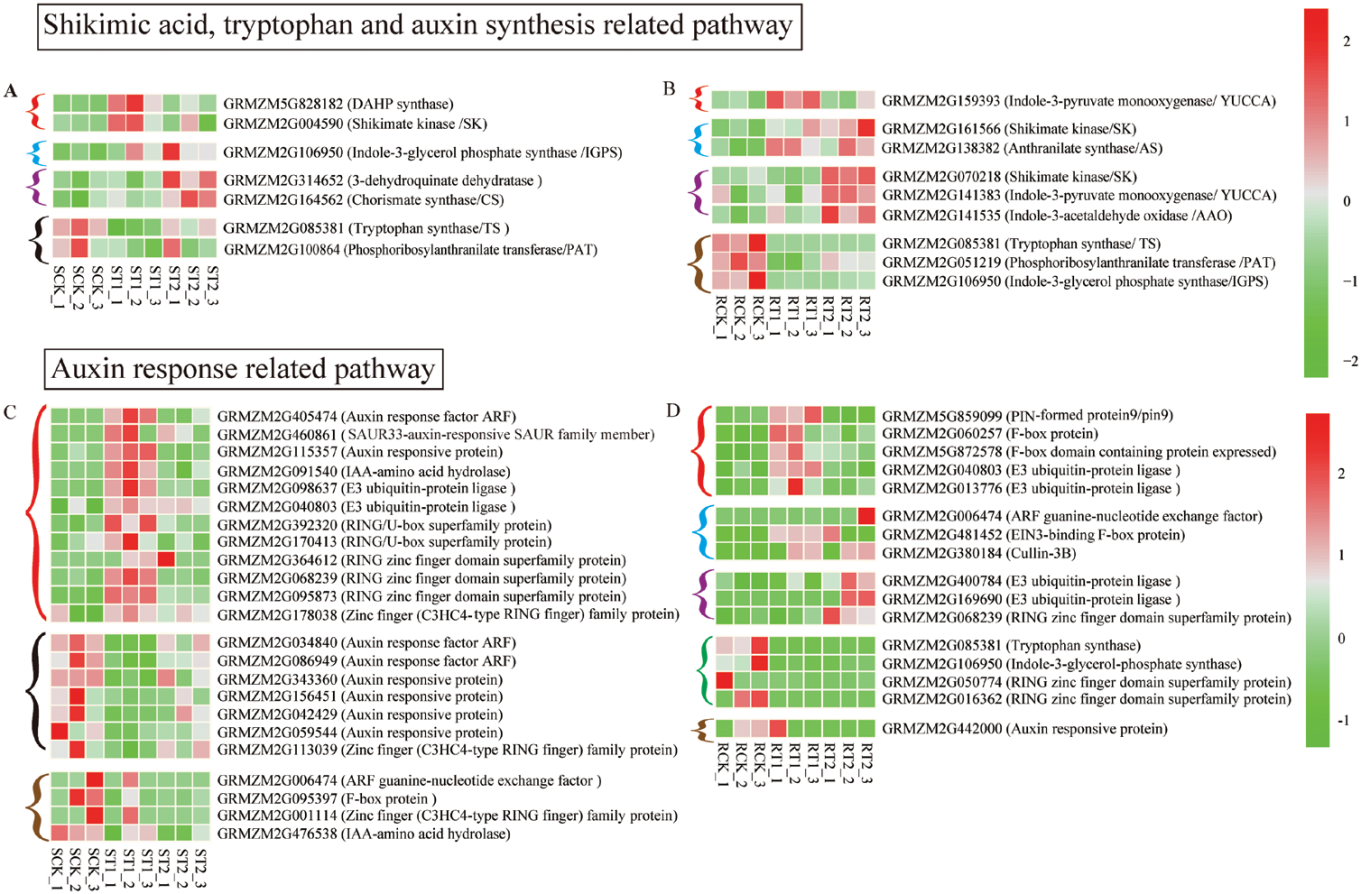
Transcriptome heat map for differentially expressed genes of the shikimic acid, tryptophan synthesis, and auxin synthesis-related pathways (A, B) and auxin response-related pathway (C, D) in the shoot and root of maize plants. SCK, ST1, and ST2 indicate the levels of gene expression in the shoot of plants exposed to sole NO_3_^–^, mixed N, and sole NH_4_^+^ supply, respectively. RCK, RT1, and RT2 indicate the levels of gene expression in the root of plants exposed to sole NO_3_^–^, mixed N, and sole NH_4_^+^ supply, respectively. Three repeats were performed for each N treatment. The color gradient illustrates the *Z*-scores of the gene expression values calculated as the mean-centered log2 (FPKM) values divided by the SD for each gene. Significant difference for each gene was defined as *P*<0.05 and log2 (FPKM) values ≥1 or ≤–1. Compared with expression in the presence of sole NO_3_^–^ supply, the expression of genes is either up-regulated by mixed N supply (red bracket), up-regulated by both mixed N and sole NH_4_^+^ supply (blue bracket), up-regulated by sole NH_4_^+^ supply (purple bracket), down-regulated by mixed N supply (black bracket), down-regulated by both mixed N and sole NH_4_^+^ supply (green bracket), or down-regulated by sole NH_4_^+^ supply (brown bracket).

The expression of key genes involved in the shikimic acid, Trp synthesis, and Trp-dependent auxin synthesis pathways was further investigated by RNA-Seq analysis (Fig. 4A, B) and confirmed by real-time quantitative PCR (RT–PCR) (Fig. S9 at Dryad). In the shoot, the genes encoding DAHP synthase (GRMZM5G828182) and shikimate kinase (GRMZM2G004590) were up-regulated under mixed N compared with sole NO_3_^–^ supply (Fig. 4A). The indole-3-glycerol phosphate synthase gene (GRMZM2G106950) was up-regulated by both mixed N and sole NH_4_^+^ supply. The genes encoding 3-dehydroquinate dehydratase (GRMZM2G314652) and chorismate synthase (GRMZM2G164562) were up-regulated only by NH_4_^+^. In the root, the gene encoding indole-3-pyruvate monooxygenase (YUCCA) (GRMZM2G159393) was up-regulated by mixed N supply (Fig. 4B). The shikimate kinase (GRMZM2G161566) and anthranilic acid synthase (GRMZM2G138382) genes were up-regulated by both mixed N and sole NH_4_^+^ supply. In addition, the shikimate kinase (GRMZM2G070218), YUCCA (GRMZM2G141383), and indole-3-acetaldehyde oxidase (GRMZM2G141535) genes were up-regulated in NH_4_^+^-treated plants.

The expression of genes involved in auxin responsiveness was further investigated (Figs 4C, D; Fig. S9 at Dryad). In the shoot, several genes encoding auxin response proteins were up-regulated by the mixed N supply: the auxin response factor (GRMZM2G405474), auxin-responsive protein (GRMZM2G460861; GRMZM2G115357), E3 ubiquitin protein (GRMZM2G098637; GRMZM2G040803) and RING protein-related genes (GRMZM2G392320; GRMZM2G170413; GRMZM2G364612; GRMZM2G068239; GRMZM2G095873 and GRMZM2G178038). Interestingly, the sole NH_4_^+^ supply did not increase the expression of genes related to the auxin response. In the roots, the PIN protein gene (GRMZM5G859099), F-box protein genes (GRMZM2G060257; GRMZM5G872578), and E3 ubiquitin protein genes (GRMZM2G040803; GRMZM2G013776) were up-regulated by the mixed N supply. The ARF guanine-nucleotide exchange factor gene (GRMZM2G006474), F-box protein gene (GRMZM2G481452), and Cullin protein gene (GRMZM2G380184) were up-regulated by both the mixed N and sole NH_4_^+^ supply. The E3 ubiquitin protein genes (GRMZM2G400784; GRMZM2G169690) and RING protein gene (GRMZM2G068239) were also up-regulated by NH_4_^+^ supply.

### Nitrogen assimilation

**Fig. 5.**
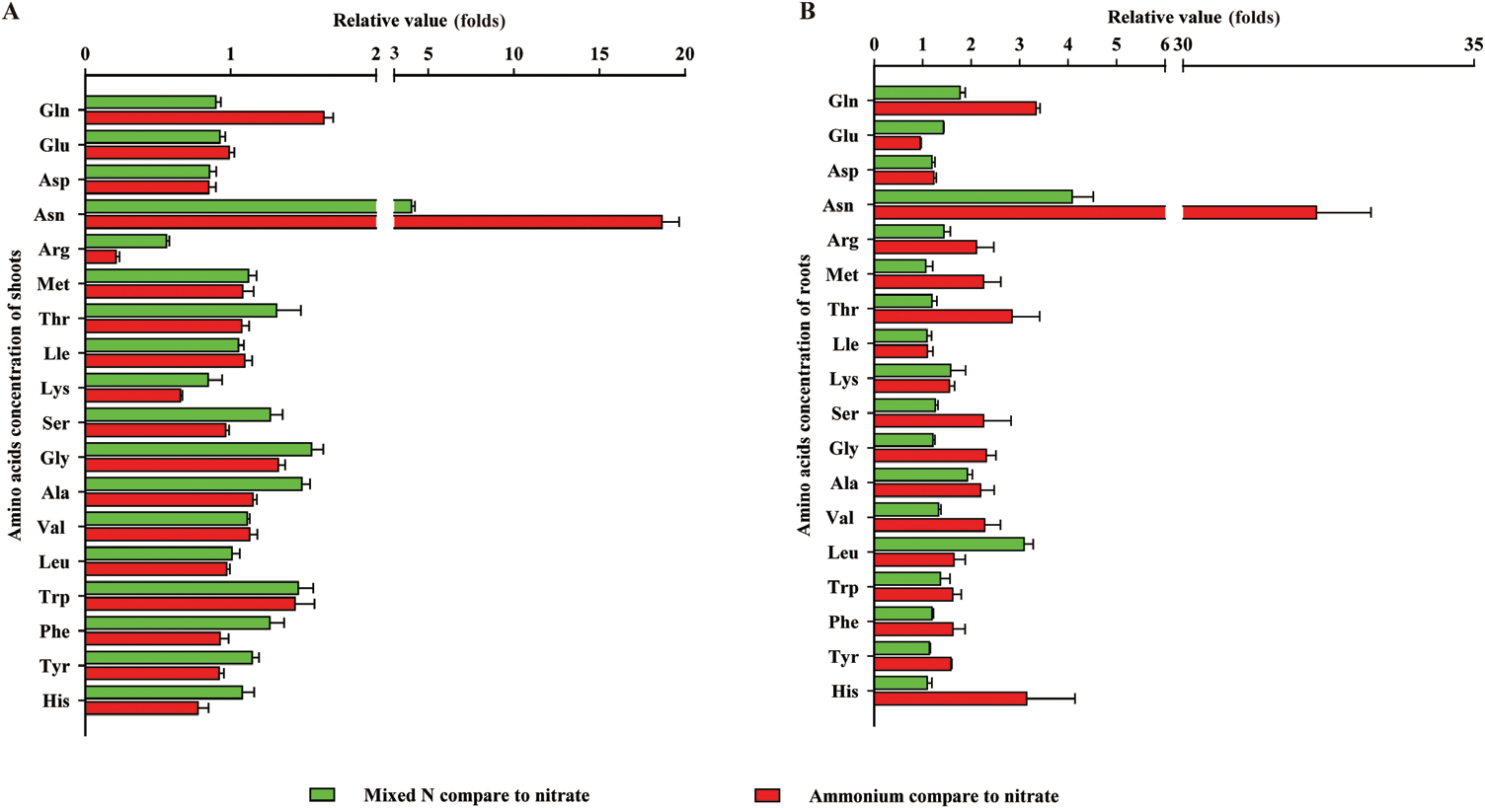
Relative values of amino acid concentrations in the shoot (A) and root (B) of maize grown in the presence of a mixed N supply and sole NH_4_^+^ supply compared with sole NO_3_^–^ supply. Values are mean ±SE (*n*=7).

Compared with the effects of sole NO_3_^–^ supply, adding NH_4_^+^ increased NH_4_^+^ and decreased NO_3_^–^ concentration in the shoot and root (Fig. S10 at Dryad). The mixed N supply reduced the concentration of Asp (0.85-fold), increased that of Asn (4.02-fold), and had no effect on the concentrations of Gln and Glu in the shoot. In the root, the mixed N supply increased the concentration of all four of these amino acids, especially Asn (4.09-fold). Under the sole NH_4_^+^ supply, the concentration of Asn greatly increased in both shoot (18.64-fold) and root (32.29-fold). Gln also increased in the shoot (1.64-fold) and root (3.34-fold). Asp decreased in the shoot (0.85-fold) but increased in the root (1.23-fold). The Glu concentration in shoot and root was not significantly affected by NH_4_^+^ treatment (Fig. 5). Considering other amino acids, the mixed N supply increased the concentrations of Ser, Gly, Ala, Trp, Phe, and Try (1.15- to 1.49-fold), decreased the concentration of Arg (0.56-fold) in shoot, and had little effect on the other amino acids measured. The mixed N supply increased the concentrations of Arg, Met, Ser, Gly, Ala, Val, Leu, Trp, Phe, and Tyr (1.06- to 3.09-fold) in the root, and had little effect on other amino acids. Sole NH_4_^+^ supply increased the concentrations of Gly, Ala, and Trp (1.15- to 1.44-fold) and decreased Arg and His (0.21- to 0.77-fold) in the shoot. In the root, the NH_4_^+^ supply increased the concentration of all amino acids measured (1.10- to 3.14-fold) (Fig. 5).

**Fig. 6.**
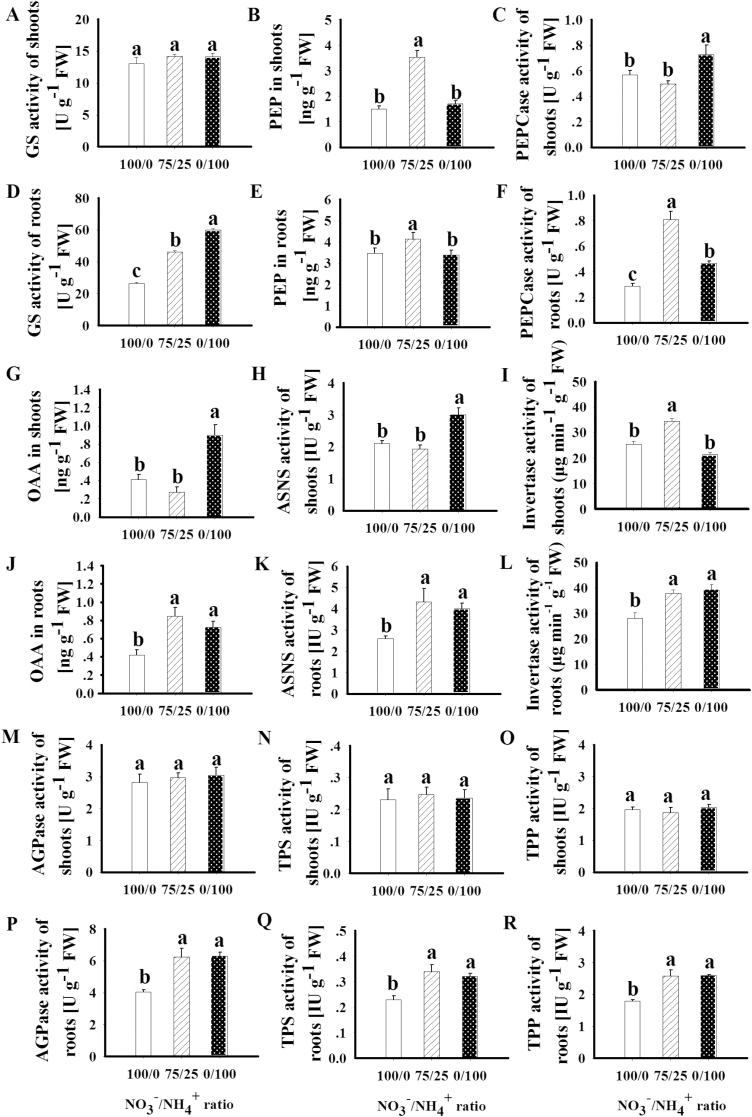
Effects of different forms of N on GS activity (A, D), PEP concentration (B, E), PEPCase activity (C, F), OAA concentration (G, J), ASNS activity (H, K), invertase activity (I, L), AGPase activity (M, P), TPS activity (N, Q), and TPP activity (O, R) in the shoot and root of maize plants. 100/0, sole NO_3_^–^ supply; 75/25, 75%/25% NO_3_^–^/NH_4_^+^; 0/100, sole NH_4_^+^ supply. Values are mean ±SE (*n*=6). Significant differences between treatments (*P*<0.05) are indicated with different letters.

The activity of glutamine synthase (GS) was not affected by the different forms of N in the shoot, but in the root it was increased 1.75-fold by the mixed N supply, and 2.28-fold by the NH_4_^+^ supply (Fig. 6A, D). Correspondingly, the expression of two GS genes (GRMZM2G024104; GRMZM2G046601) was up-regulated in the root by NH_4_^+^ supply (Fig. S11B at Dryad). NH_4_^+^ supply inhibited the expression of the nitrate reductase (GRMZM5G878558) and nitrite reductase (GRMZM2G102959; GRMZM2G079381) genes in the shoot and root (Fig. S11 at Dryad).

### PEP and downstream organic acid metabolism

PEP is a central player linking Trp-dependent auxin synthesis, respiratory metabolism via PK-mediated pyruvate synthesis, and PEPCase-mediated OAA synthesis and downstream Asn synthesis via ASNS. Compared with the sole NO_3_^–^ supply, the mixed N supply increased PEP concentrations in the shoot and root by 2.35- and 1.19-fold, respectively (Fig. 6B, E). The mixed N supply also increased PEPCase activity, OAA concentration, and ASNS activity in the root, but had no such effects in the shoot (Fig. 6). In the shoot, the mixed N supply increased the expression of the genes encoding PEPCase (GRMZM2G083841, Zm00001d024980), pyruvate decarboxylase (AC197705.4_FG001), Acyl-CoA synthetase (GRMZM2G120539), malate transporter (GRMZM2G436593), and Asp and Asn-related protein (GRMZM2G468857). In the root, the mixed N supply increased the expression of the genes encoding the 3-phosphoglycerate transporter (AC203985.4_FG001; GRMZM2G104942), triose phosphate isomerase (GRMZM2G146206), malate transporter (GRMZM2G089396; GRMZM2G094860), PK (GRMZM2G178047), and ASNS (GRMZM2G053669; GRMZM2G078472) (Fig. S11 at Dryad).

Compared with NO_3_^–^ supply, the NH_4_^+^ supply did not affect the PEP concentration in the shoot and root (Fig. 6B, E). NH_4_^+^ increased PEPCase activity, OAA concentration, and ASNS activity in both the shoot and root (1.28- to 1.72-fold; Fig. 6). In the shoot, the expression of the PEPCase (Zm00001d024980) and pyruvate decarboxylase (AC197705.4_FG001) genes was up-regulated by NH_4_^+^ supply (Fig. S11A at Dryad). In the root, NH_4_^+^ supply increased the expression of the ASNS (GRMZM2G053669; GRMZM2G078472), malate transporter (GRMZM2G089396; GRMZM2G094860), PK (GRMZM2G178047), and glutamate dehydrogenase (GRMZM2G427097) genes (Fig. S11B at Dryad).

### Sugar metabolism

**Fig. 7.**
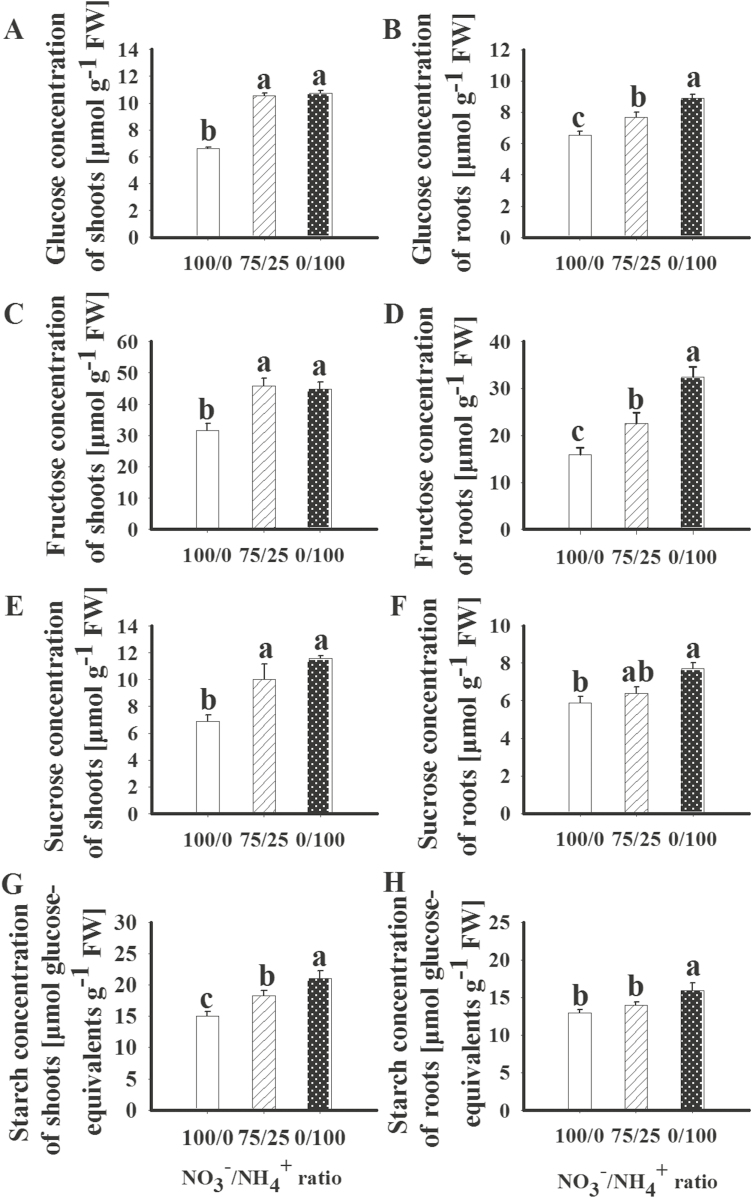
Concentrations of glucose (A, B), fructose (C, D), sucrose (E, F), and starch (G, H) in the shoots and roots of maize plants grown in the presence of different forms of N. 100/0, sole NO_3_^–^ supply; 75/25, 75%/25% NO_3_^–^/NH_4_^+^; 0/100, sole NH_4_^+^ supply. Values are mean ±SE (*n*=7). Significant differences between treatments (*P*<0.05) are indicated with different letters.

Compared with the sole NO_3_^–^ supply, the mixed N supply increased the shoot concentrations of glucose (1.60-fold), fructose (1.44-fold), sucrose (1.45-fold), and starch (1.22-fold). In the roots, the mixed N supply increased the glucose and fructose concentrations by 1.18- and 1.43-fold, respectively, but had no effect on sucrose and starch (Fig. 7). The hydrolysis of sucrose into glucose and fructose is catalyzed by invertase (Figueroa *et al.*, 2016). The mixed N supply increased invertase activity by1.61-fold in the shoot and 1.35-fold in the root (Fig. 6I, L). The sole NH_4_^+^ supply increased the concentrations of glucose, fructose, sucrose, and starch in both the shoot and root (1.31- to 2.04-fold; Fig. 7). NH_4_^+^ increased invertase activity by 1.39-fold in the root but had no such effect in the shoot (Fig. 6).

**Fig. 8.**
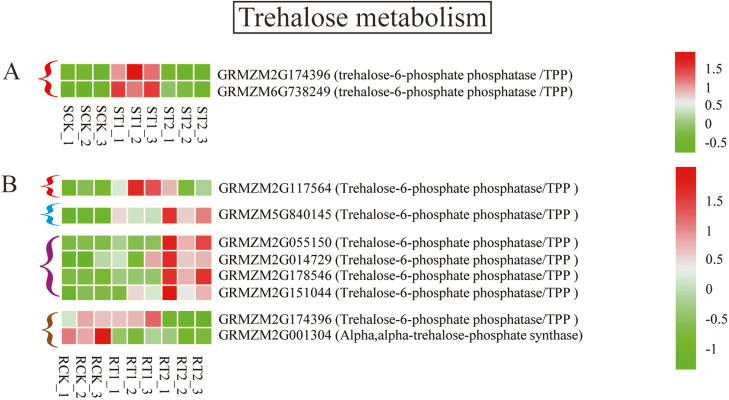
Transcriptome heat map for differentially expressed genes of trehalose metabolism in the shoot (A) and root (B). SCK, ST1, and ST2 indicate the levels of gene expression in the shoot of plants exposed to sole NO_3_^–^, mixed N, and sole NH_4_^+^ supply, respectively. RCK, RT1, and RT2 indicate the levels of gene expression in the root of plants exposed to sole NO_3_^–^, mixed N, and sole NH_4_^+^ supply, respectively. Three repeats were performed for each N treatment. The color gradient illustrates the *Z*-scores of the gene expression values calculated as the mean-centered log2 (FPKM) values divided by the SD for each gene. Significant difference for each gene was defined as *P*<0.05 and log2 (FPKM) values ≥1 or ≤–1. Compared with expression in the presence of sole NO_3_^–^ supply, the expression of genes is either up-regulated by mixed N supply (red bracket), up-regulated by both mixed N and sole NH_4_^+^ supply (blue bracket), up-regulated by sole NH_4_^+^ supply (purple bracket), or down-regulated by sole NH_4_^+^ supply (brown bracket).

The precursor for both starch and T6P synthesis is G1P. G1P is converted into G6P and then to T6P by the enzymes TPS and TPP, or into starch by AGPase. In the shoot, the activity of TPS, TPP, and AGPase did not differ among the three N treatments (Fig. 6M–O). In the root, both mixed N supply and NH_4_^+^ supply increased the activity of TPS, TPP, and AGPase, by 1.40- to 1.56-fold. This finding is largely consistent with the higher concentrations of G1P and T6P in roots supplied with mixed N or NH_4_^+^ (Fig. 2B). Compared with the NO_3_^–^ supply, the mixed N supply up-regulated the expression of trehalose-6-phosphate phosphatase genes in the shoot (GRMZM2G174396; GRMZM2G179349) and in the root (GRMZM2G117564, GRMZM5G840145). The NH_4_^+^ supply also up-regulated the expression of trehalose-6-phosphate phosphatase genes (GRMZM5G840145, GRMZM2G055150, GRMZM2G014729, GRMZM2G178546, and GRMZM2 G151044) in the root (Fig. 8).

## Discussion

### Mixed nitrogen supply increases both carbon source and carbon utilization

In the experimental conditions used in the present study, with 1 mM N concentration and controlled solution pH, the NH_4_^+^-treated plants did not show any symptoms of NH_4_^+^ toxicity and grew better than the NO_3_^–^-treated plants. In the study of Prinsi *et al.* (2018), NH_4_^+^-fed plants grew less well than NO_3_^–^-fed plants, possibly because the authors grew maize plants in 5 mM NH_4_^+^ and without stabilizing the medium pH. It has been suggested that the inhibitory effect of NH_4_^+^ on plant growth is related to lower sugar levels (Ganmore and Kafkafi, 1983; Schortemeyer *et al.*, 1997). In our study, the NH_4_^+^-fed plants had higher sugar levels than the NO_3_^–^-fed plants, which may explain their superior growth.

It has been reported that NH_4_^+^ treatment increases photosynthesis in various crops (Fuhrer and Erismann, 1984; Raab and Terry, 1994; Bowler, 1996; Claussen and Lenz, 1999; Guo *et al.*, 2001). In the present study, compared with sole NO_3_^–^ supply, both mixed N and sole NH_4_^+^ supply increased the photosynthetic rate (Fig. 1D), as well as the concentrations of glucose, fructose, sucrose, and starch in shoots (Fig. 7). However, shoot and root biomass, as well as N accumulation, were increased in plants treated with the mixed N supply to a much greater extent than in plants supplied with NH_4_^+^ alone (Fig. 1). Similar results were reported by George *et al.* (2016). As found previously (Tabatabaei *et al.*, 2008; Zhu *et al.*, 2014; Xu *et al.*, 2017), leaf area was increased by the mixed N supply, but not by sole NH_4_^+^ supply. The greater leaf growth in plants supplied with mixed N indicates a stronger sink for C and N utilization. Accordingly, invertase activity was 1.61-fold higher while the starch concentration was 0.87-fold lower in the shoots of plants treated with mixed N relative to plants supplied with NH_4_^+^, indicating that C is more utilized in plants supplied with mixed N. On the other hand, the lower concentrations of Gln and Asn suggest more utilization of N in the plants treated with mixed N compared with those receiving sole NH_4_^+^ supply (Fig. 5; Fig. S12 at Dryad).

Excessive accumulation of starch may impair chloroplast function (Neales and Incoll, 1968). The results of the present study suggest that the redundant C in NH_4_^+^-fed plants is used to assimilate inorganic N into N storage forms such as Gln and Asn, thus increasing the concentrations of these amino acids (Fig. 5; Fig. S12 at Dryad). This process can avoid the negative feedback effect of nitrogenous compounds on NO_3_^–^ or NH_4_^+^ uptake (Sabermanesh *et al.*, 2017; Plett *et al.*, 2018; Tegeder and Masclaux-Daubresse, 2018). The transcriptomic analysis indicates that mixed N supply significantly promotes the NO_3_^–^ transporter GRMZM2G044851 (Zm*NRT1.5a*), as well as potassium channel genes (GRMZM2G156255), which may improve NH_4_^+^ absorption (Table S7 at Dryad).

Recent studies have shown that T6P is an important regulator in plant metabolism (Yadav *et al.*, 2014; Figueroa and Lunn, 2016). Chemically increasing the level of T6P in wheat plants greatly increases yield via regulation of sugar allocation and utilization (Griffiths *et al.*, 2016). Figueroa *et al.* (2016) found that increasing the T6P level in Arabidopsis can stimulate N assimilation, thereby diverting sucrose for amino acid synthesis. The higher T6P concentration in roots under NH_4_^+^ supply observed in the present study may be used as a signal to induce more Gln and Asn synthesis. Increasing the level of T6P in plants can promote the synthesis of starch in Arabidopsis (Kolbe *et al.*, 2005; Yadav *et al.*, 2014; Figueroa *et al.*, 2016). Consistently, in this study, the concentrations of T6P and starch in roots under NH_4_^+^ supply were 4.5- and 1.23-fold higher than their respective levels in roots under NO_3_^–^ supply; by contrast, in roots under mixed N supply, T6P was increased by only 1.87-fold and there was little effect on the starch level (Fig. S12 at Dryad). Higher levels of T6P may serve as a signal to promote starch synthesis and drive more flow of sucrose from shoot to root, leading to greater synthesis of Asn and Gln in the root.

### Mixed N supply increases auxin via the shikimic acid pathway

Auxin regulates leaf development by controlling the initiation of leaf primordia (Reinhardt *et al.*, 2000), vascular development (Sieburth, 1999), and leaf cell division and enlargement (Keller, 2017). In this study, the IAA concentration was highest under mixed N supply in both the shoot and root, suggesting that IAA may play a role in promoting plant growth. Accordingly, the metabolomics data confirmed that the metabolites involved in IAA synthesis are increased in the presence of mixed N supply.

Trp, the precursor for auxin synthesis (D’Mello, 2015), is synthesized mainly through the shikimic acid pathway, which in plants begins with the binding of PEP and D-erythrose-4-phosphate (Maeda and Dudareva, 2012). In this study, the metabolomics analysis identified that Trp was increased by the mixed N supply. Transcriptomic analysis further indicated that the expression of the genes encoding DAHP synthase (GRMZM5G828182), indole-3-glycerol phosphate synthase (GRMZM2G106950), and shikimate kinase (GRMZM2G004590) were all up-regulated in plants treated with the mixed N supply (Fig. 4). Taking these findings together, it can be postulated that the mixed N supply increased the expression of genes related to Trp and IAA synthesis, thus increasing the level of IAA (Fig. 3), which plays a role in promoting shoot and root growth, so building a large sink for C and N.

The plant response to auxin involves a series of auxin signal transductions involving the auxin receptor TIR1, auxin response factor (ARF) protein family, and the Aux/IAA protein family (Tan *et al.*, 2007). In this study, the expression of genes encoding E3 (GRMZM2G098637; GRMZM2G040803) and RING (GRMZM2G364612; GRMZM2G170413; GRMZM2G170413; GRMZM2G364612; GRMZM2G068239; GRMZM2G095873; GRMZM2G178038) proteins were significantly up-regulated in shoots under mixed N supply; this up-regulation may mediate Aux/IAA ubiquitination and improve the expression of *ARF* (GRMZM2G405474) (Fig. 4). In addition to the IAA/Auxin and ARF pathway, SMALL AUXIN UP RNAs (SAURs) are also involved in the early auxin response (Druege *et al.*, 2016). SAURs are transcriptionally induced by auxin in different species. In the shoot, SAURs control cell expansion, probably via targeting PP2C.D phosphatases, which act as inhibitors of plasma membrane H^+^-ATPase (Ren and Gray, 2015). In the present study, *SAUR* (GRMZM2G460861) was significantly up-regulated in shoots under mixed N supply, further supporting the role of the auxin pathway in promoting shoot and root growth. Interestingly, there was no difference between NH_4_^+^ and NO_3_^–^ supply in terms of effect on the expression of auxin response-related genes.

A previous study in tobacco showed that NO_3_^–^ promotes leaf expansion by increasing root-to-shoot transport of CTK (Wahl and Ryser, 2000). In barley, however, NH_4_^+^ increases the level of zeatin riboside in roots compared with NO_3_^–^ supply (Samuelson and Larsson, 1993). In our study, the CTK concentrations in the shoot were similar between plants supplied with NO_3_^–^ and NH_4_^+^. It is possible that the effect of the form of N on CTK synthesis and/or transport varies among different species. We found a low concentration of CTK in shoots under mixed N supply, and therefore cannot explain the considerable leaf growth that occurred under mixed N supply. Among the other plant hormones investigated, BR and GA3 are reported to affect the rate of cell division by regulating the expression of genes involved in the cell division process (Gutierrez, 2009; Avramova *et al.*, 2015; Mu *et al.*, 2018). In our study, however, we did not find that the changes in the levels of these hormones in the shoot were consistent with the changes in leaf area under the three different N treatments (Fig. S8 at Dryad).

## Conclusion

**Fig. 9.**
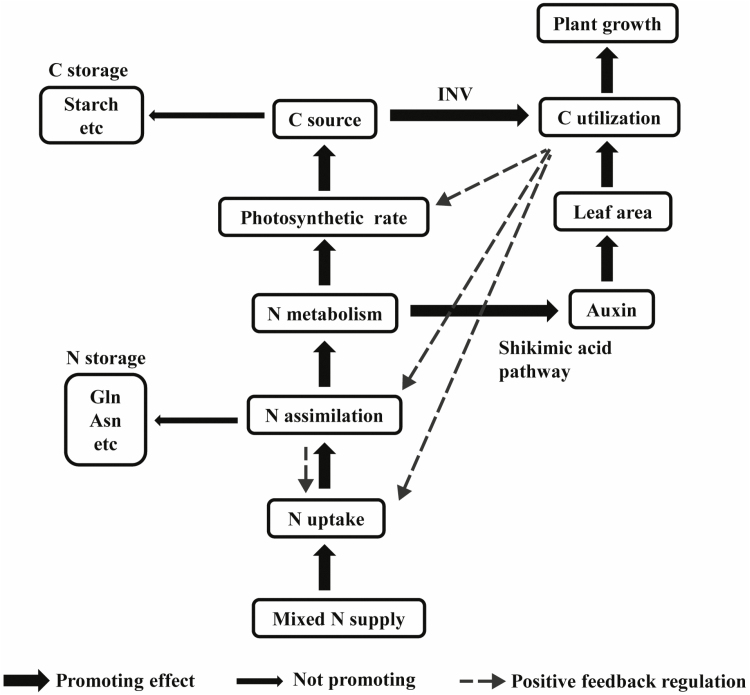
A model explaining the promoting effect of mixed N form on maize growth. The thick arrows represent promoting effect. The thin arrows represent not promoting. The dotted arrows represent positive feedback regulation. On one hand, mixed N supply increases C source by promoting photosynthesis rate. On the other hand, mixed N supply enhances auxin synthesis via shikimate pathway so as to increased leaf area. As a result, C utilization is enhanced, which exerts a positive feedback regulation effect on photosynthetic rate, N assimilation, and N uptake.

Optimizing the source–sink relationship is an important way to increase crop yield (Paul *et al.*, 2017). The use of a mixed N supply not only increases C supply by promoting photosynthesis, but also enhances C utilization by stimulating leaf growth. The mixed N supply stimulates Trp-dependent IAA synthesis via the shikimate pathway and tryptophan pathway, and up-regulates the auxin response pathway so as to increase leaf growth. The larger leaf area that results under mixed N supply exerts positive feedback, enhancing photosynthesis and N uptake (Fig. 9). Although using NH_4_^+^ supply also results in a higher photosynthetic rate compared with the use of sole NO_3_^–^, it has lower potential in terms of IAA synthesis compared with the mixed N supply. As a result, under sole NH_4_^+^ treatment, C is not efficiently used for shoot and root growth; instead, the surplus C flows to the synthesis of storage metabolites such as starch, Asn, and Gln, possibly via the T6P signaling pathway.

## Data deposition

The following data are available at Dryad Digital Repository: https://dx.doi.org/10.5061/dryad.cd57c84.

Table S1. Primers used in qRT–PCR analysis of genes related to IAA synthesis and the auxin response pathway.

Table S2. Differential metabolites in the shoot identified by LC-MS secondary mass spectrometry.

Table S3. Differential metabolites in the root identified by LC-MS secondary mass spectrometry.

Table S4. Transcriptome sequencing data statistics and quality evaluation.

Table S5. Differentially expressed genes related to photosynthesis in shoots.

Table S6. Differentially expressed genes of ion transportation in shoots.

Table S7. Differentially expressed genes of ion absorption and transportation in roots.

Fig. S1. Total chlorophyll concentration and shoot and root N concentration as affected by N forms.

Fig. S2. Total ion current LC-MS chromatogram.

Fig. S3. Principal component analysis of the differential metabolites.

Fig. S4. Score of partial least square discriminant analysis.

Fig. S5. Differentially expressed genes statistics under mixed N supply or sole ammonium supply compared to sole nitrate supply in shoot and root.

Fig. S6. Gene ontology for differentially expressed genes under mixed N supply compared to nitrate supply, and under ammonium supply compared to nitrate supply, in shoots.

Fig. S7. Gene ontology for differentially expressed genes under mixed N supply compared to nitrate supply, and under ammonium supply compared to nitrate supply, in roots.

Fig. S8. CTK, BR, GA3, JA, and SA in the shoot and root of plants grown in different N forms.

Fig. S9. qRT–PCR confirmation of key genes related to IAA synthesis via the shikimic acid pathway, and auxin response pathways in the shoot.

Fig. S10. Free nitrate and ammonium concentration in the shoot and root of maize supplied with different N forms.

Fig. S11. Transcription heat map of differentially expressed genes related to amino acid and organic acid metabolism in the shoot and root.

Fig. S12. Diagram showing the differential effects of mixed N supply and ammonium supply on key metabolic pathways compared to sole nitrate supply.

Raw data deposition: Transcriptome raw data from maize (Zhengdan958) are uploaded to the SRA database, SRA accession: PRJNA506798. Metabolomics LC-MS raw data from maize (Zhengdan958) are available at Dryad Digital Repository: https://doi.org/10.5061/dryad.cd57c84.

## Abbreviations:

AGPase: ADP-glucose pyrophosphorylase
ASNS: asparagine synthase
BR: brassinosteroid
C: carbon
CTK: cytokinin
G1P: glucose monophosphate
GA3: gibberellin 3
GO: gene ontology
GS: glutamine synthase
IAA: auxin
JA: jasmonic acid
N: nitrogen
NH_4_^+^: ammonium
NO_3_^−^: nitrate
OAA: oxaloacetate
PEP: phosphoenolpyruvate
PEPCase: phosphoenolpyruvate carboxylase
PK: pyruvate kinase
SA: salicylic acid
T6P: trehalose hexaphosphate
TPP: trehalose-phosphate phosphatase
TPS: trehalose-phosphate synthase
YUCCA: indole-3-pyruvate monooxygenase
